# The dose-dependent impact of γ-radiation reinforced with backscatter from titanium on primary human osteoblasts

**DOI:** 10.1080/26415275.2023.2209116

**Published:** 2023-05-16

**Authors:** Lisa Printzell, Janne Elin Reseland, Nina Frederike Jeppesen Edin, Hanna Tiainen, Jan Eirik Ellingsen

**Affiliations:** a Department of Prosthodontics, Faculty for Dentistry, Institute of Clinical Dentistry, University of Oslo, Oslo, Norway; b Department of Biomaterials, Faculty for Dentistry, Institute of Clinical Dentistry, University of Oslo, Oslo, Norway; c Department of Physics, Faculty of Mathematics and Natural Science, University of Oslo, Oslo, Norway

**Keywords:** Radiotherapy, radiation backscatter, osteogenic differentiation, titanium implants, osseointegration

## Abstract

In head and neck cancer patients receiving dental implants prior to radiotherapy, backscatter from titanium increases the radiation dose close to the surface, and may affect the osseointegration. The dose-dependent effects of ionizing radiation on human osteoblasts (hOBs) were investigated. The hOBs were seeded on machined titanium, moderately rough fluoride-modified titanium, and tissue culture polystyrene, and cultured in growth- or osteoblastic differentiation medium (DM). The hOBs were exposed to ionizing γ-irradiation in single doses of 2, 6 or 10 Gy. Twenty-one days post-irradiation, cell nuclei and collagen production were quantified. Cytotoxicity and indicators of differentiation were measured and compared to unirradiated controls. Radiation with backscatter from titanium significantly reduced the number of hOBs but increased the alkaline phosphatase activity in both types of medium when adjusted to the relative cell number on day 21. Irradiated hOBs on the TiF-surface produced similar amounts of collagen as unirradiated controls when cultured in DM. The majority of osteogenic biomarkers significantly increased on day 21 when the hOBs had been exposed to 10 Gy, while the opposite or no effect was observed after lower doses. High doses reinforced with backscatter from titanium resulted in smaller but seemingly more differentiated subpopulations of osteoblasts.

## Introduction

Radiation therapy, in combination with surgical interventions, is the predominant treatment modalities for head and neck cancer patients [1]. Infection preventive regimens prior to radiotherapy (RT) comprise multiple tooth extractions for most of the patients. The result is an edentulous or partially edentulous situation, which in combination with radiation-induced side effects such as xerostomia and hypersensitive oral mucosa considerably impairs the patient’s oral function and aesthetics. In the attempt to improve the oral health-related quality of life in these patients, osseointegrated dental implants (ODIs) are indispensable in terms of an adequate prosthetic rehabilitation [2–5].

Ionizing radiation causes detrimental biologic effects on the bone within the area of treatment [6,7], thereby altering the osseous healing capacity, and ultimately, the chance of a successful osseointegration of dental implants [8,9]. Several systematic reviews have reported unpredictable ODI survival rates in irradiated jaws [10–14]. As seen in these systematic reviews, the *prognostic factors* for long-term ODI survival are numerous; however, the total dose of radiation delivered to the specific implant site is essential for implant survival. Moreover, the timepoint of implant installation relative to the RT also seems to affect the outcome. Implants placed in the mandible exhibit higher survival rates than those placed in the maxilla, and similarly, implants placed in natural bone show superior survival compared to implants placed in grafted bone [10–14]. Furthermore, hyperbaric oxygen (HBO) therapy prior to implant insertion may enhance the prognosis by improving microvascular perfusion, and thus, the osseous healing capacity in irradiated bone [15,16].

In unirradiated bone, certain implant surface configurations have shown to promote and accelerate the osseointegration process [17–20]. For instance, fluoride-modified titanium is reported to improve osteoblast (OB) differentiation and adhesion, and thus, enhance the formation of a direct bone-to-implant contact [21–24]. The clinical relevance of surface modifications of dental implants inserted in *irradiated* jaws is seldom described. However, implants with a turned surface have shown to fail almost three times more often than implants with a roughened surface, especially when placed in the maxilla [25].

The optimal timing of dental implant surgery in head and neck cancer patients treated with RT has not yet been defined. Data evaluated in a review from 2013 supported installation of implants either 14 days or more before RT (primary placement) or between 6 and 24 months after RT (secondary placement) [26]. Advantages of the primary placement protocol include the potential for osseointegration in unirradiated bone, no need for pre-surgical adjunctive HBO therapy, and a significantly faster prosthetic rehabilitation for the patients [27,28]. Secondary placement will consequently leave the patient without teeth or fixed dentures during the post-surgical and post-RT healing period and may delay the rehabilitation process for 1–2 years. Already in 2007, a systematic review showed promising outcomes after primary placement, with no significant difference in failure rates between implants placed prior to RT (5.4%) or after RT (3.2%) [29]. Since then, several clinical trials have reported positive results on the primary placement protocol [30–33]. However, precautions must be taken as radiation backscatter from titanium has been shown to escalate the radiation dose to the cells growing on the surface with more than 40% [34]. Fortunately, backscattered radiation from titanium is of a very short range and cells at a distance of 1 mm from the surface receive only a 15% higher radiation dose due to backscatter [34].

Validated protocols for primary placement of dental implants are necessary in order to increase the potential for restoring oral function and aesthetics in head and neck cancer patients at an early phase. To achieve this goal, knowledge about how radiation backscatter from titanium influences the cells involved in the osseointegration process is fundamental. Osteoblasts play an essential role in bone regeneration and remodeling, and thus, in the osseointegration of dental implants [35]. While a previous report showed that the escalated radiation dose caused by backscatter from titanium did not influence the response of human mesenchymal stem cells significantly [34], OBs have demonstrated a radiation dose-dependent reduction in cell attachment and proliferation [36,37]. However, *in vitro* studies investigating the effects of irradiation on human OBs cultured on titanium are very limited.

The aim of this study was to evaluate the dose-dependent effects of ionizing γ-irradiation on primary human osteoblasts (hOBs) cultured on different titanium surfaces compared to on plastic to assess the potential effects of radiation backscatter on the cells.

## Materials and methods

### Test surfaces

Titanium disks with a diameter of 6.25 and height of 1.95 mm were used as test specimens. The disks were treated in two different ways to obtain two different titanium surfaces. The minimally rough non-modified titanium (Ti) surface was a commercially pure (cp) grade 2 titanium, manufactured at mechanical and electronic workshops (Domus Medica, University of Oslo, Oslo, Norway). The machined surface of this as-produced sample disk was shiny with circular grooves from the grinding. These disks were washed in trichloroethylene for 15 min, then absolute ethanol (100%) in an ultrasonic bath for 15 min, before being sterilized in an autoclave at 135 °C for 20 min. The fluoride-modified titanium (TiF) was a commercially available implant surface (Osseospeed, Dentsply Sirona, Mölndal, Sweden) consisting of a moderately rough cp grade 4 titanium, grit-blasted with titanium dioxide (TiO_2_) microparticles, followed by an initial etching in concentrated nitric acid (HNO_3_) and sodium hydroxide (NaOH), respectively, before a final etching step in 0.1 M hydrofluoric acid (HF). The disks were pre-mounted on carriers in sealed containers and sterilized by β-irradiation, according to the manufacturer’s protocol (Dentsply Sirona, Mölndal, Sweden). The third test surface was the floor of the tissue culture polystyrene (TCP) well-plates. The test surfaces were imaged using a single-reflex digital camera (Nikon D3200 equipped with a Nikon 105 mm f/2.8D AF Micro Nikkor macro lens, Minato City, Japan) using fixed imaging settings and lighting conditions. Images were adjusted for white balance and exposure using Adobe Lightroom Classic. Scanning electron microscopy (SEM) of the test surfaces was performed using TM-3030 table-top SEM (Hitachi, Tokyo, Japan), imaging the samples at 15 kV acceleration voltage using backscattered electrons. For this purpose, TCP samples were sputtered with a thin conductive gold layer (2–3 nm) prior to SEM imaging. To quantify the microscale topography of the Ti, TiF and TCP surfaces, an optical image profilometer (Sensofar S neox, Barcelona, Spain) with a 150× objective was used. Three non-overlapping areas on three independent sample surfaces were analyzed per sample group (*n* = 3). Surface roughness (*S*_a_) of the test surfaces was calculated from profilometry data. Wettability of the test surfaces was determined by measuring the water contact angle (WCA) on these surfaces using an optical contact angle measuring system (OCA 20, DataPhysics Instruments, Filderstadt, Germany). The contour of a 1 µl sessile drop of milliQ water on the sample surface was recorded at room temperature and the contact angle was determined using ellipse fitting for 30°≤ WCA ≤ 90° and tangent fitting for WCA < 30°. Three non-overlapping areas on three independent sample surfaces were analyzed per sample group (*n* = 3). The measurements were performed on freshly prepared samples.

### Cells and experimental design

Titanium disks were prepared and placed in 96-well plates (Sigma-Aldrich, St. Louis, MO, USA). Primary human OBs (batch no. 0000426160; Lonza, Walkersville, MD, USA) in passage 5 were seeded at confluence (×10^4^ cells per well) on Ti, TiF and TCP (*n* = 6), and placed in an incubator at 37 °C in a humid atmosphere of 5% CO_2_. Cells cultured on the same surface were divided into two medium groups, receiving either OB growth medium (GM) (*n* = 3) consisting of OB basal medium (C-27010) supplemented with Osteoblast Growth Medium SupplementMix (C-39615) containing l-glutamine, gentamicin sulfate-amphotericin (GA) and 10% fetal bovine serum (FBS) (Promocell, Heidelberg, Germany), or osteogenic differentiation medium (DM) (*n* = 3) prepared by supplementing the GM with hydrocortisone (200 nM), beta-glycerophosphate (10 mM) and ascorbic acid (0.28 mM) (Sigma-Aldrich, St. Louis, MO, USA).

The hOBs were γ-irradiated at The Norwegian Radium Hospital, Oslo University Hospital, 24 h after initial seeding. All cell plates including the controls were sealed in plastic during transport and irradiation to avoid contamination of bacteria. The cell plates were also packed in styrofoam during the transport and kept in an incubator at the Radium Hospital before and after the radiation exposures to keep the temperature stable. Nominal doses of 2, 6 and 10 Gy (excluding the backscatter contribution) were achieved by calculating the duration of each irradiation, considering the source-to-plate distance of 70 cm and the decay of the ^60^Co source (Theratron 780-C, MDS Nordion, Ontario, Canada). The selection of radiation doses was based on existing literature on similar experiments on stem cells and OBs [34,38,39]. To maintain the cell medium temperature at 37 °C, a hollow perspex plate transfused with circulating pre-heated water (Grant Instruments, Cambridge, England) was used throughout the irradiation procedure. Dose measurements were achieved by thermoluminescence dosimetry (TLD) (TLD-100; Harshaw TLD Bicrom, Solon, OH, USA).

Unirradiated cells on the same surfaces, exposed to exactly the same conditions (GM or DM) and handling procedures as irradiated cells, were used as control. The medium (GM or DM) was changed twice per week throughout the follow-up period and 24 h before sampling at day 1 (acute effects) and day 21 (late effects). The medium samples were stored at −20 °C prior to analyses. Collected data from irradiated cells were normalized to the data of unirradiated controls on the same surface, and in the same type of medium, collected at the same timepoint. The experimental set-up was repeated three times, using commercially available primary human OBs from the same donor (Lonza, Walkersville, MD, USA).

### Evaluation of cell number and morphology

Twenty-one days post-irradiation, hOBs on the Ti- and TiF-surfaces were washed with phosphate-buffered saline (PBS) and fixed with 4% paraformaldehyde (PFA). The number of cell nuclei, collagen content and intracellular actin was assessed by fluorescence microscopy imaging using an upright Leica SP8 confocal laser scanning microscope with air objective lens HC APO CS 10×/0.40 (Leica, Wetzlar, Germany). The samples were stained with mouse anti-collagen IA primary antibody (Abcam, Cambridge, UK) and goat anti-mouse Alexa 488 (Invitrogen, Eugene, OR, USA), and co-stained with phalloidin Alexa 568 (Thermo Scientific, Carlsbad, CA, USA) and Hoechst 33342 (Sigma-Aldrich, St. Louis, MO). ImageJ software (National Institutes of Health and the Laboratory for Optical and Computational Instrumentation, Bethesda, MD) was used for image processing and analysis [40]. The number of cells attached to the titanium surfaces was estimated by counting cell nuclei from four representative areas on each individual coin (*n* = 3) using automatic thresholding and particle analysis in ImageJ (Bethesda, MD). The fluorescence intensities of collagen and actin were also quantified from the same fields of view. While data collected from the medium samples represent the hOBs activity for the last 24 h, the content of collagen and actin displays cell production during the entire follow-up period of 21 days.

Cells on TCP were regularly monitored using an Olympus IX70 inverted microscope (Olympus, Shinjuku, Tokyo, Japan) throughout the post-irradiation follow-up period of 21 days. To determine the amounts of cells growing on TCP on day 21, images from 6 to 8 representative areas per sample (*n* = 3) were used to estimate a mean percentage of cell confluence on the surface. It was not possible to assess collagen and actin on the transparent TCP-surface due to the fluorescence imaging technique used.

### Cytotoxicity

Lactate dehydrogenase (LDH) levels, as an indicator of radiation-induced cell membrane leakage, were measured in the cell culture medium (*n* = 9) on day 1, 7, 14 and 21 post-irradiation using a cytotoxicity detection kit (Roche Diagnostics, Mannheim, Germany). The medium was changed 24 h prior to sampling. Spectrophotometry was used to determine LDH activity by measuring the absorbance at 490 nm using an ELx800 Absorbance Reader (BioTek Instruments, Winooski, VT, USA).

### Mineralization and signs of differentiation

Alkaline phosphatase (ALP) activity in the cell culture medium (*n* = 9) on day 1 and day 21, as an indicator of cell differentiation, was quantified in the medium samples by a cleavage reaction of *p*-nitrophenyl phosphate (pNPP) (Sigma-Aldrich, St. Louis, MO). The reaction was stopped after 30 min in the dark at room temperature and spectrophotometry was then used to measure the soluble yellow end-product at 405 nm absorbance using an ELx800 Absorbance Reader (BioTek Instruments, Winooski, VT). In parallel to the samples, a standard curve was created using calf intestinal ALP (CIAP; Promega, Madison, WI, USA) with standards ranging from 0 to 6000 pM. Collected data from day 21 were adjusted to the relative cell number.

On day 21 post-irradiation, cells on all surfaces (Ti and TiF; *n* = 6, TCP; *n* = 9) were fixed with 4% PFA and stained with 1% alizarin red (pH 4.2) for 5 min [41]. Mineralization was quantified by extracting the alizarin red deposition with 10% cetyl pyridinium chloride (Sigma-Aldrich, St. Louis, MO) at room temperature and then measuring at 562 nm using an ELx800 Absorbance Reader (BioTek Instruments, Winooski, VT, USA) as previously described [42].

### Osteogenic biomarkers

Selected biomarkers secreted from the hOBs to the cell culture medium (*n* = 9) were quantified by multianalyte profiling using the Luminex 200 system (Luminex, Austin, TX, USA). Concentrations of dickkopf-related protein 1 (DKK-1), interleukin-6 (IL-6), osteoprotegerin (OPG), osteocalcin (OC) and osteopontin (OPN), were measured using the HBNMAG-51K Human Bone kit (EMD Millipore, Billerica, MA, USA) according to the manufacturer’s protocol. Minimum detectable concentrations varied between 0.4 and 68.5 pg/ml for the selected markers. The data were analyzed by xPONENT 3.1 software (Luminex, Austin, TX, USA) for measurement of samples harvested on day 1 and day 21 post-irradiation.

### Statistical analysis

The effects of irradiation (2, 6 or 10 Gy) on hOBs growing on three different surfaces (Ti, TiF and TCP) were analyzed using Sigmaplot (V 13.0 for Windows; Systat, Chicago, IL, USA). The effects of irradiation were normalized to unirradiated cells on the same surfaces, cultured in the same type of medium, and with the same duration of incubation. Data on ALP and osteogenic biomarkers were adjusted to the relative cell number on day 21. Differences between groups were assessed using one-way ANOVA followed by pairwise multiple comparison using the Holm-Sidak method. The significance level was set to *p* values ≤0.05. All data are presented as mean values ± standard error of the mean (SEM), and as a percentage of the values obtained for unirradiated controls.

## Results

### Surface characteristics

**Figure 1.**
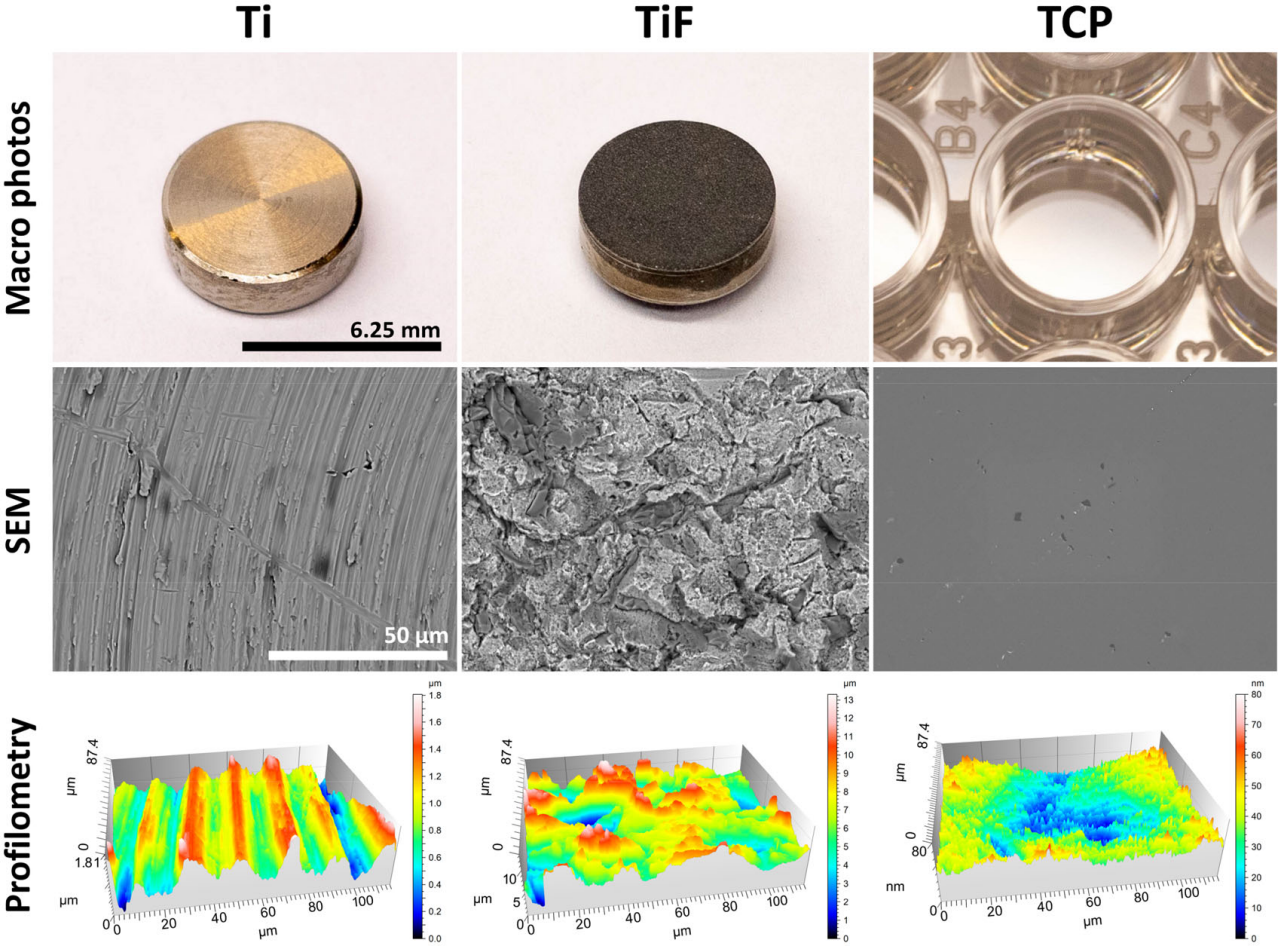
Macro photos, scanning electron microscopy images and 3D surface topography of the three different test surfaces. Surface roughness (*S*_a_) of the test surfaces was calculated from the obtained profilometry data, Ti: 0.25 ± 0.01 µm; TiF: 1.94 ± 0.16 µm; TCP: 9.34 ± 0.21 nm (*n* = 3).

The three tested sample surfaces and their surface topography are illustrated in Figure 1. The Ti samples consisted of machined titanium surfaces characterized by concentric grooves of approximately 5 µm wide and 0.5 µm deep (*S*_a_: 0.25 ± 0.01 µm), whereas the TiF samples had isotropically rough grit blasted surfaces with considerably higher roughness (*S*_a_: 1.94 ± 0.16 µm). The control surface (TCP) was smooth on both micro- and nanoscale (*S*_a_: 9.34 ± 0.21 nm). The wettability of the different surfaces was determined by measuring the WCA on the three different surfaces. The TiF surface had the highest wettability with WCA of 9.4 ± 2.3°, while the WCA of Ti (57.9 ± 5.5°) and TCP (72.3 ± 5.1°) was significantly higher (*p* < 0.05), indicating lower wettability on these surfaces. No statistically significant difference was detected between the Ti and TCP (*p* > 0.05).

### Evaluation of cell number and cytotoxicity

**Figure 2.**
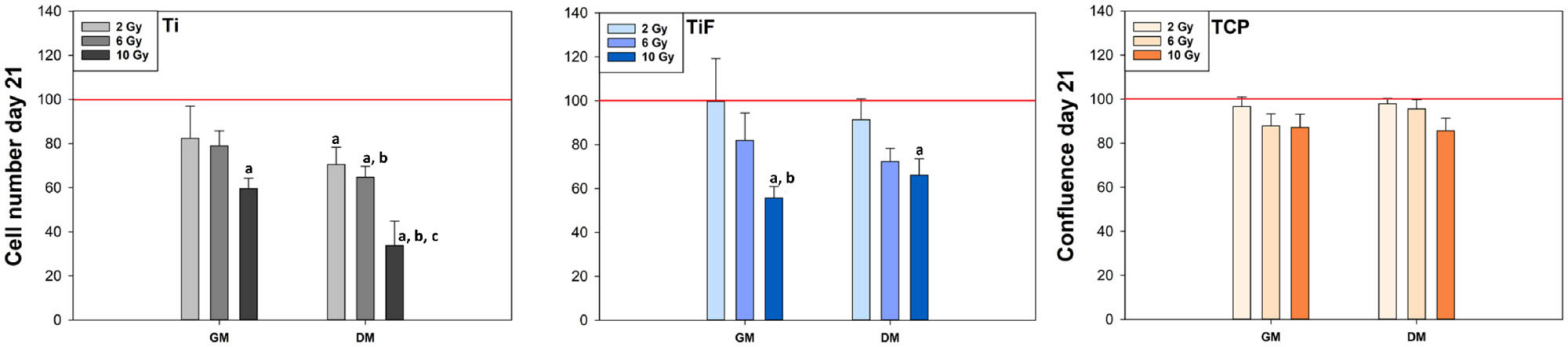
The number of irradiated human osteoblasts (hOBs) cultured in growth medium (GM) or differentiation medium (DM) on minimally rough machined titanium (Ti) and moderately rough fluoride-modified titanium (TiF), and the relative confluence of hOBs on tissue culture polystyrene (TCP), 21 days post-irradiation. Data represent mean values ± standard error of the mean (SEM) from three different experiments (*n* = 9) and are presented as % of unirradiated controls (red line) on the same surfaces (Ti, TiF or TCP) and in the same type of culture medium (GM or DM). The significance level is set to *p* values ≤0.05 toward control (red line) (a), toward 2 Gy (b) and toward 6 Gy (c).

**Figure 3.**
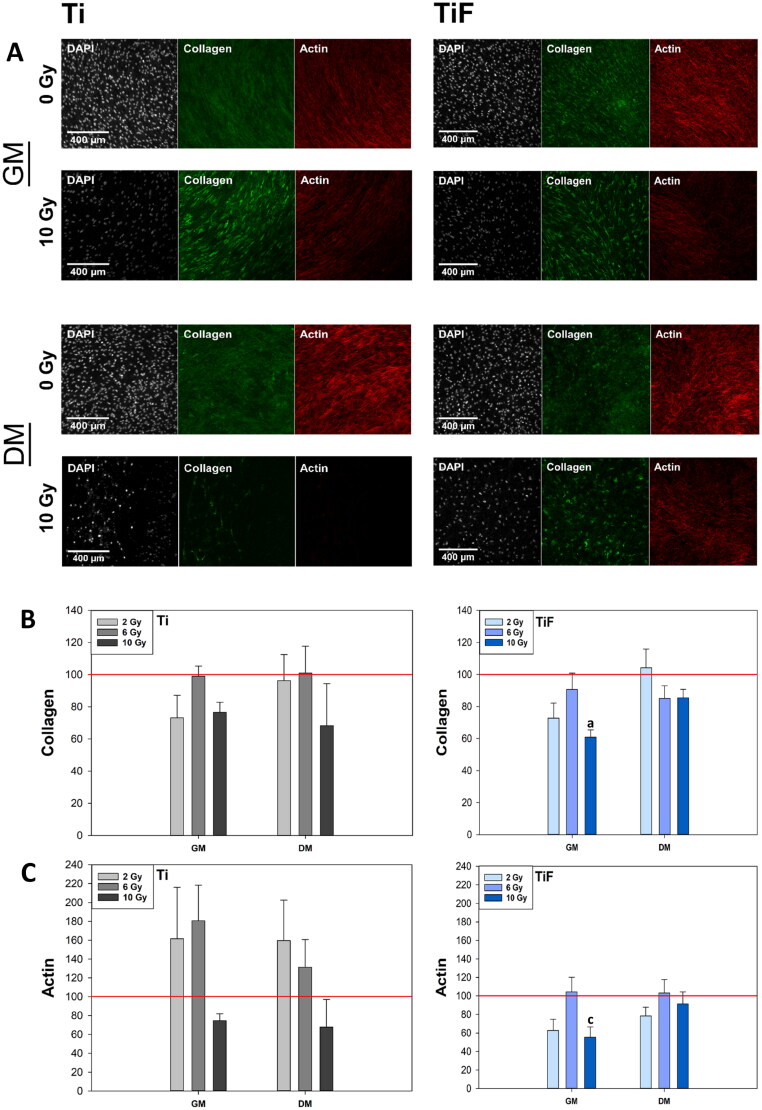
Confocal images from one of the experiments. Morphology of cell nuclei, collagen and actin production of hOBs cultured for 21 days in growth medium (GM) (*n* = 3) or differentiation medium (DM) (*n* = 3) on minimally rough machined titanium (Ti) and moderately rough fluoride-modified titanium (TiF), after no irradiation and after 10 Gy (A). Quantification of collagen (B) and actin (C) production of hOBs cultured for 21 days in GM (*n* = 3) or DM (*n* = 3), on Ti and TiF, after single doses of 2, 6 and 10 Gy. The data represent mean values ± standard error of the mean (SEM) from three different experiments and are presented as % of unirradiated controls (red line) on the same surfaces (Ti or TiF) and in the same type of culture medium (GM or DM). The significance level is set to *p* values ≤ 0.05 toward control (a), and toward 6 Gy (c).

A radiation dose-dependent reduction in the number of hOBs on the Ti- and the TiF-surfaces was observed irrespective of the type of medium used (Figure 2). On the Ti-surface, irradiation with 10 Gy reduced the number of hOBs cultured in GM to 60%, and in DM to 34%, and on the TiF-surface to 56 and 66%, respectively. A slight dose-dependent reduction in hOBs was also observed on TCP, but this reduction was not found to be significant. The number of *unirradiated* control cells 21 days post-irradiation was not significantly affected by surface modification (Ti/TiF) or type of cell culture medium (GM/DM) as illustrated in Figure 3(A).

Lactate dehydrogenase activity measured in the cell culture medium on days 1, 7, 14 and 21 was not significantly affected by irradiation, as shown in supporting information (Figure S1). No temporal changes in LDH concentration from unirradiated hOBs were found on any of the test surfaces.

### Quantification and morphology of collagen and actin

The type of cell culture medium used (GM or DM) did not affect the amount of collagen produced by *unirradiated* hOBs (Figure 3(A)). On the TiF-surface, irradiation with 10 Gy significantly reduced the collagen production from hOBs cultured in GM, while irradiation did not affect the collagen production when the hOBs were cultured in DM on the same surface (Figure 3(B)). Morphologically, the collagen rods produced by hOBs in GM appeared to be arranged in a laminar manner, while the collagen from hOBs in DM appeared as a more irregular structure (Figure 3(A)).

Unirradiated hOBs cultured in DM on the Ti-surface exhibited a 26% higher actin content than those cultured in GM, whereas the actin production in unirradiated cells on the TiF-surface was not influenced by type of culture medium (Figure 3(A)). Irradiation with 2 and 6 Gy induced a higher actin production in cells cultured on the Ti-surface independent of medium; however, this was not statistically significant (Figure 3(C)). On the TiF-surface, irradiation with 10 Gy significantly reduced the actin production in hOBs cultured in GM (*p* ≤ 0.05) when compared to hOBs irradiated with 6 Gy, while the production was similar to controls when the cells were cultured in DM. The actin morphology reflects the cell shape, being rounder when cells are under differentiation. The actin filaments of hOBs cultured in DM showed a more circumferential spreading onto the surface, as seen prior to differentiation (Figure 3(A)).

### Mineralization and signs of differentiation

**Figure 4.**
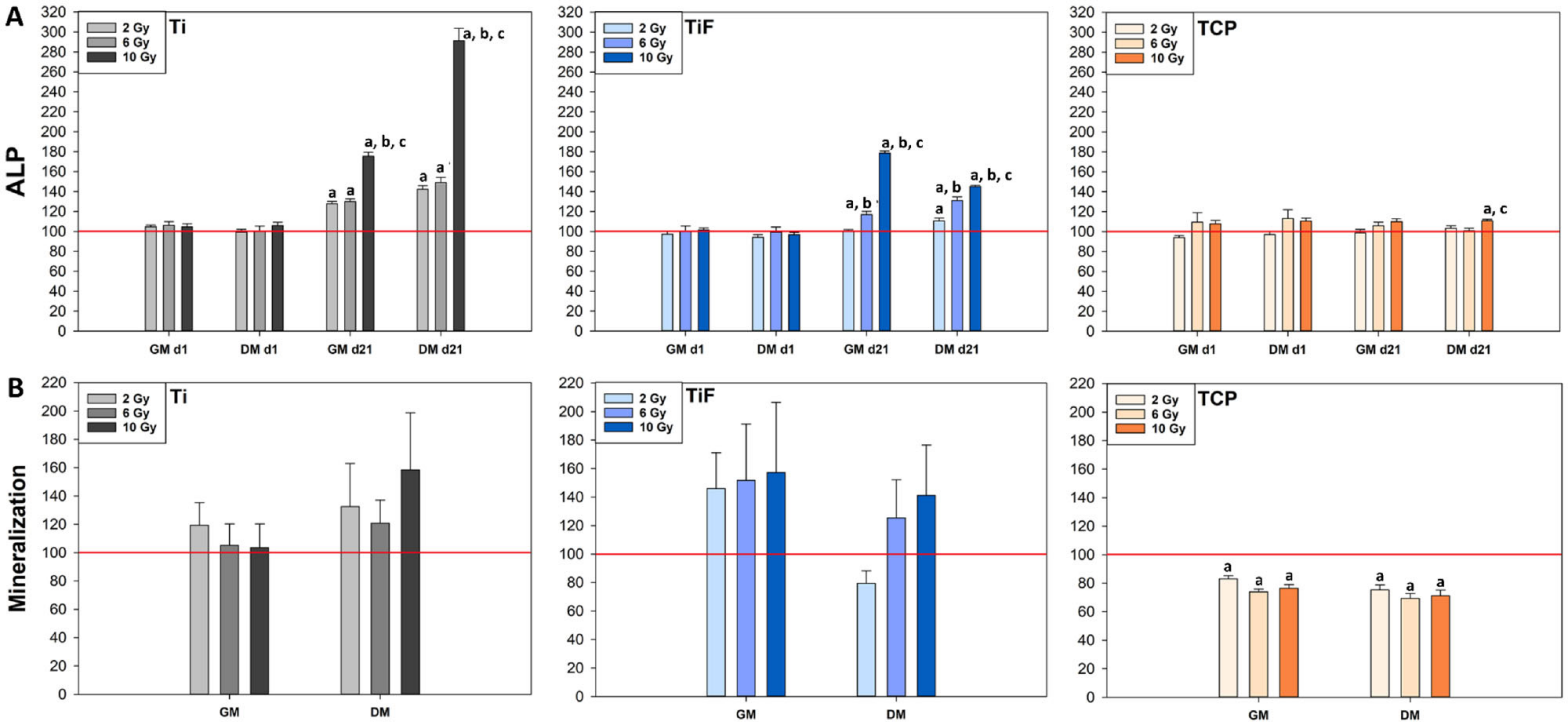
Alkaline phosphatase (ALP) activity measured in growth medium (GM) (*n* = 9) and differentiation medium (DM) (*n* = 9) of human osteoblasts (hOBs) on days 1 and 21 post-irradiation (A), and the effects of radiation on the mineral deposition of hOBs after 21 days of culture (B) on minimally rough machined titanium (Ti) (*n* = 6), moderately rough fluoride-modified titanium (TiF) (*n* = 6), and tissue culture polystyrene (TCP) (*n* = 9), after single doses of 2, 6 and 10 Gy. The data represent mean values ± standard error of the mean (SEM) from three different experiments and are presented as % of unirradiated controls (red line) on the same surface (Ti, TiF or TCP) and timepoint. The significance level is set to *p* values ≤ 0.05 toward control (a), toward 2 Gy (b) and toward 6 Gy (c).

The ALP activity in the cell culture medium was adjusted to the relative cell number on day 21 for each individual sample, and the results for the radiation doses tested are presented in Figure 4(A). Irradiation enhanced the ALP activity from the remaining hOBs at day 21 post-irradiation in a dose-dependent manner, especially from hOBs cultured on the Ti and the TiF-surfaces.

Irradiation significantly reduced the mineral deposition of hOBs growing on TCP, independent of radiation dose and type of cell culture medium. In contrast, mineral deposition of cells on the titanium surfaces seemed to increase with radiation, but due to a wide spread in data this was not statistically significant (Figure 4(B)).

### Osteogenic biomarkers

**Figure 5.**
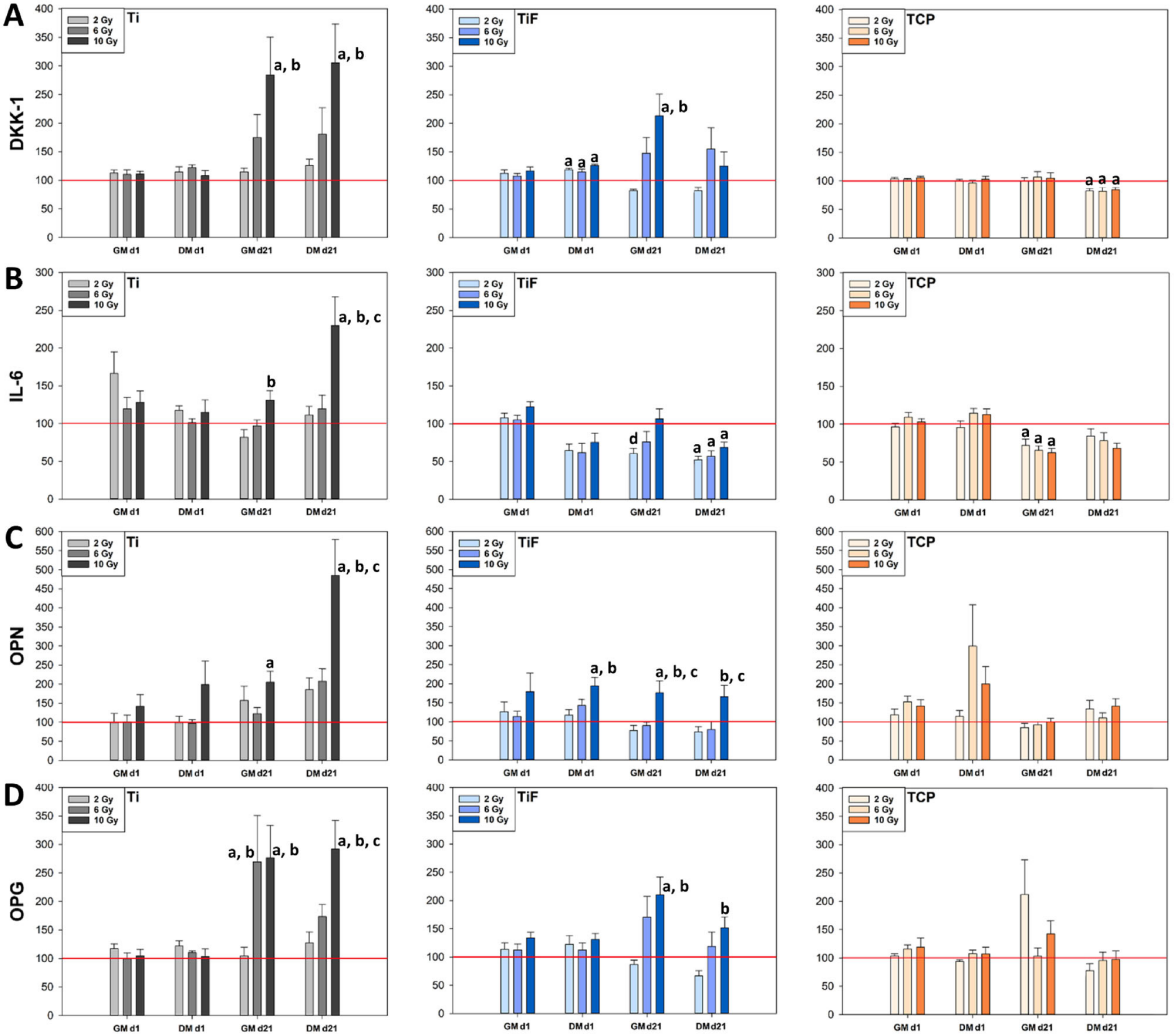
Secretion of osteogenic biomarkers; dickkopf-related protein (DKK-1) (A), interleukin-6 (IL-6) (B), osteopontin (OPN) (C) and osteoprotegerin (OPG) (D) to the cell culture medium (GM and DM) measured on days 1 and 21 post-irradiation, from human osteoblasts (hOBs) cultured on minimally rough machined titanium (Ti) (*n* = 9), moderately rough fluoride-modified titanium (TiF) (*n* = 9) and tissue culture polystyrene (TCP) (*n* = 9) after single doses of 2, 6 or 10 Gy. The data are presented as % of unirradiated controls (red line) on the same surfaces (Ti, TiF or TCP) and timepoints. Data were adjusted to the relative cell number on day 21 and represent mean values ± standard error of the mean (SEM) from three different experiments. The significance level is set to *p* values ≤ 0.05 toward control (a), toward 2 Gy (b), toward 6 Gy (c) and toward 10 Gy (d).

Four measured osteogenic biomarkers (DKK-1, IL-6, OPN and OPG) were found in both types of cell culture medium of hOBs. The concentrations of these factors are presented in Figure S2 before the data were adjusted to the relative cell number on day 21 post-irradiation, of each of the respective surfaces (Figure 5).

Irradiation with 10 Gy significantly enhanced the levels of DKK-1 from hOBs growing on the Ti-surface when adjusted to cell number on day 21 post-irradiation, while on the TiF-surface only when the hOBs were cultured in GM. In comparison, irradiated hOBs on TCP secreted similar DKK-1 levels as unirradiated controls after all doses and days tested, except for significantly lower levels on day 21 when the cells were cultured in DM (Figure 5(A)). When adjusted to the relative cell number on day 21, irradiation with 10 Gy significantly increased the IL-6 secretion from hOBs growing on the Ti-surface. In contrast, irradiation reduced the IL-6 secretion from hOBs cultured on the TiF and the TCP-surface at day 21 post-irradiation (Figure 5(B)).

The OPN secretion significantly increased when the hOBs were irradiated with 10 Gy while cultured on the Ti and TiF-surface (Figure 5(C)).

The OPG secretion exhibited the highest concentrations on day 21 (Figure S2), and showed a significant increase after irradiation with 10 Gy on titanium (Ti and TiF) when adjusted to the relative cell number and compared to unirradiated controls (Figure 5(D)).

After irradiation with 2 and 6 Gy, most of the osteogenic biomarkers decreased or were not significantly affected.

## Discussion

The present study demonstrated that radiation backscatter from titanium significantly reduced the number of human OBs in a distinct dose-dependent manner, with the lowest number of cells found on the minimally rough Ti-surface 21 days after exposure to a 10 Gy dose of ionizing γ-irradiation. The lack of temporal changes in LDH activity throughout the observation period demonstrates that the reduced cell number is not due to an acute cytotoxic response but more likely a combination of genotoxic effects of radiation [43,44] and reduced proliferation capacity [36]. This is also consistent with the observation of Li et al. [37] who found a dose-dependent decrease in cell proliferation that was more pronounced when the irradiated OB-like cells were cultured on titanium compared to on TCP. Li et al. also found that irradiated cells cultured on rougher modified titanium showed both higher adhesion ability and collagen secretion than cells cultured on a polished titanium surface. Correspondingly, we observed a less pronounced reduction in relative number of irradiated hOBs when cultured in DM on the rougher TiF-surface compared to the machined Ti-surface (Figure 2), although the influence of this surface on collagen production was not confirmed. Nevertheless, irradiated hOBs cultured in DM on the TiF-surface produced similar amounts of collagen as unirradiated controls after all radiation doses tested. Bearing in mind the significantly lower number of cells present on the titanium surfaces at the end of the experiment, this could indicate that irradiation may enhance the collagen production of hOBs. Alternatively, irradiation may induce accelerated differentiation of the hOBs, since the production of type I collagen is one of the early signs of OBs differentiation [45].

Several reports claim that irradiation promotes the differentiation of OBs, and to a large extent, this is demonstrated by a dose-dependent increase in ALP activity [46,47]. The same trend was observed in the present study, but additionally, we showed that the ALP activity was enhanced even further when the cells were irradiated on titanium, and thus, exposed to radiation backscatter. This finding is in accordance with Ahmad et al. [36], who studied the backscatter effects from titanium on human fetal OBs (hFOB). After single doses of ionizing radiation up to 8 Gy, proliferation, differentiation and attachment to cp titanium and TCP were investigated. Comparable to our results, differentiation evaluated by ALP activity was significantly enhanced by the highest dose, while proliferation and attachment decreased with dose and time after irradiation. All these radiation-induced effects were more pronounced when the hFOBs were cultured on cp titanium, and thus, the radiation doses were enhanced by backscatter.

Independent of the type of medium used (GM or DM), the ALP activity was significantly higher for *irradiated* hOBs on titanium compared to unirradiated controls, when adjusted to the relative cell numbers on day 21. This finding may indicate that the supplemented factors in the DM were not the only osteogenic components. As proposed in previous reports, differentiation-stimulating elements may also include irradiation [36,37,44–48] titanium surface properties [49–51], and growth arrest caused by cell confluence [52]. There is a possibility that OB differentiation was initiated by cell-to-cell contact in this study, since the cells were seeded at confluence. However, this would also affect the unirradiated control cells. Modifications of the commercially used TiF-surface did not seem to enhance OB differentiation either, based on the parameters evaluated in this study. Moreover, the low mineral deposition and ALP activity detected in hOBs cultured on TCP indicate that radiation reinforced with backscatter from titanium is more likely to be the additional differentiation-stimulating factor in this study.

By adjusting the data obtained from the medium samples to the relative cell number on day 21 post-irradiation, we found that the titanium surfaces with the lowest number of cells seemed to comprise the most active hOBs in terms of osteogenesis-related proteins detected in the medium. This is consistent with the results implying that radiation backscatter from titanium reduces the number of hOBs but stimulates the differentiation process. Although several reports support a radiation dose-dependent reduction in OB proliferation [36,37,48], it is noteworthy that about 60% of all OBs undergo programmed cell death during the differentiation process toward mature bone cells [53].

The understanding that radiation backscatter most likely reduces the number of viable hOBs on a dental implant surface, and that surrounding hMSCs become less active or senescent following RT, challenges previous findings that demonstrate good results after primary placement [30–33,54]. In a clinical situation, where the physiological environments are far more complex, cells located close to a dental implant surface will contribute to the osseointegration process by cell-signaling and promoting cell migration to the wound site. In that context, cells with strong signaling capacity and expression of osteogenesis-related proteins may compensate for a lower number of cells on the implant surface by stimulating cells in the periphery to migrate, differentiate and participate in the bone healing [35]. After all, the alternative treatment to *primary placement* of dental implants is *secondary placement* which, as mentioned earlier, display unpredictable outcomes [10–14] as well as substantial delay in the rehabilitation process. In an animal model using Brazilian minipigs, RT in general was found to have a negative effect on peri-implant bone regeneration, but implants placed 15 days before RT showed better results than implants placed 3 months after RT. The authors concluded that dental implants in head and neck cancer patients should be placed before RT or simultaneously during ablative tumor surgery [54].

One notable limitation to the present study is the discrepancy between the image processing of the titanium samples compared to the TCP samples. The inverted microscope used for the transparent TCP wells excluded the possibility of counting cell nuclei, or quantifying collagen and actin in the same way as we did with the confocal laser scanning microscope images of the titanium surfaces. Instead, an estimation of the relative cell confluence on TCP was made before irradiated hOBs were compared to unirradiated controls. However, a confluent cell layer observed on TCP may also include some non-viable cells. Nonetheless, if we estimated false high amounts of cells on TCP in this study, it would not directly influence the data obtained from the Ti and the TiF-surfaces. Instead, the cell response from irradiated cells on TCP would have been increased, approaching the results obtained from the titanium surfaces, and thus, diminishes the negative effects of an implant.

The objective of this study was to investigate the dose-dependent effects of ionizing radiation on primary human OBs, and by culturing the cells on two different titanium surfaces as well as TCP (no implant), assess the potential effects of radiation backscatter. In conclusion, we found that the surviving subpopulations of hOBs 21 days after exposure to high doses (10 Gy) of radiation reinforced with backscatter seem to consist of fewer but more active and differentiated cells compared to unirradiated controls. However, hOBs irradiated with low therapeutic doses (2 Gy) of ionizing irradiation were not markedly affected. Although this information is obtained in an *in vitro* study, clinicians may find it valuable when considering primary placement of dental implants in patients planned for RT.

## Supplementary Material

Supplemental Material

## Data Availability

The datasets generated and/or analyzed during the current study are available from the corresponding author on reasonable request.
