# Supplementary material for: The dose-dependent impact of γ-radiation reinforced with backscatter from titanium on primary human osteoblasts
Source: Biomater Investig Dent. 2023 May 16;10(1):2209116. doi: 10.1080/26415275.2023.2209116 (PMC10190184; doi:10.1080/26415275.2023.2209116)
Supplement: Supplemental Material [file IABO_A_2209116_SM6164.docx]

The dose-dependent impact of γ-radiation reinforced with backscatter from titanium on primary human osteoblasts

**Supplemental material**

*Lisa Printzell^1*^, Janne Elin Reseland^2^, Nina Frederike Jeppesen Edin^3^, Hanna Tiainen^2^,*

*Jan Eirik Ellingsen^1^*

^1^Department of Prosthodontics, Institute of Clinical Dentistry, Faculty for Dentistry, University of Oslo, Oslo, Norway

^2^Department of Biomaterials, Institute of Clinical Dentistry, Faculty for Dentistry, University of Oslo, Oslo, Norway

^3^Department of Physics, Faculty of Mathematics and Natural Science, University of Oslo, Oslo, Norway

*Corresponding author: [lisa.printzell@odont.uio.no](mailto:lisa.printzell@odont.uio.no)

**Table of contents**

[Lactate dehydrogenase activity S3](#_Toc123310359)

[Figure S1 S3](#_Toc123310360)

[Concentrations osteogenic biomarkers S4](#_Toc123310361)

[Figure S2. S4](#_Toc123310362)

# Lactate dehydrogenase activity


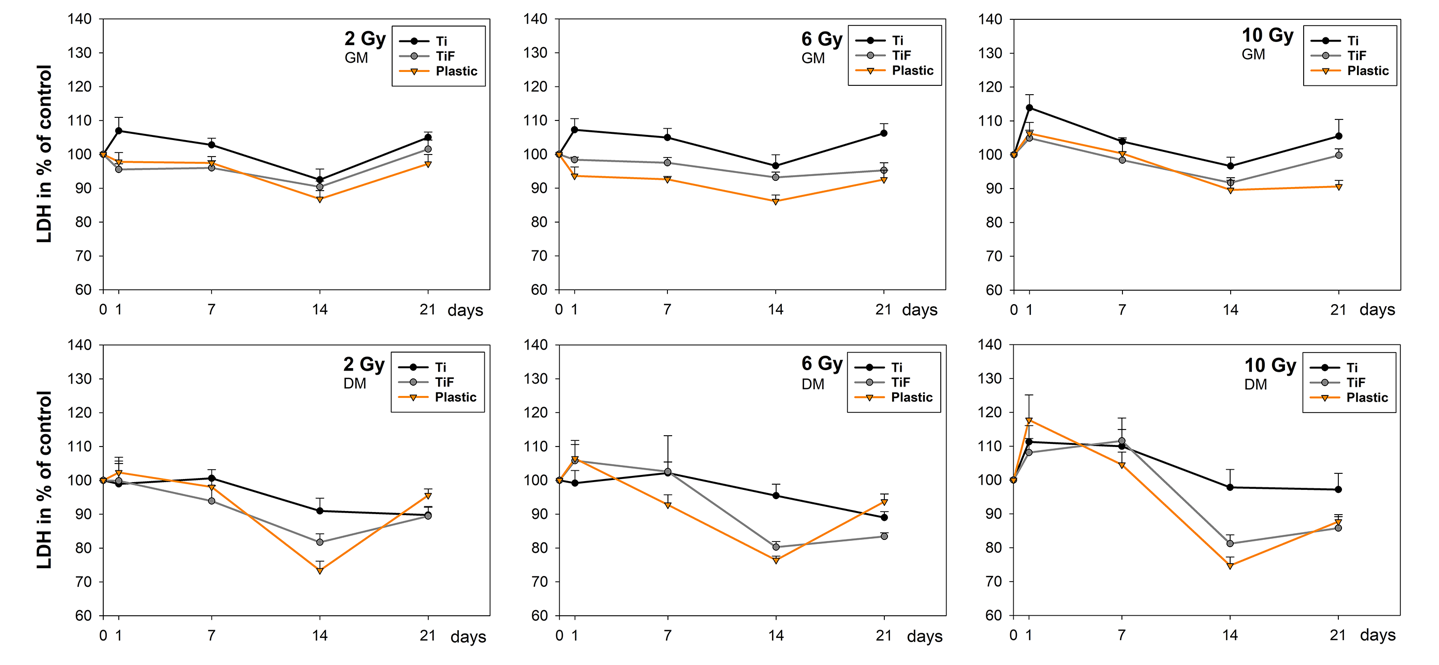


Figure S1. Temporal changes in LDH activity measured in the cell-culture medium at day 1, 7, 14 and 21 post-irradiation.

# Concentrations osteogenic biomarkers


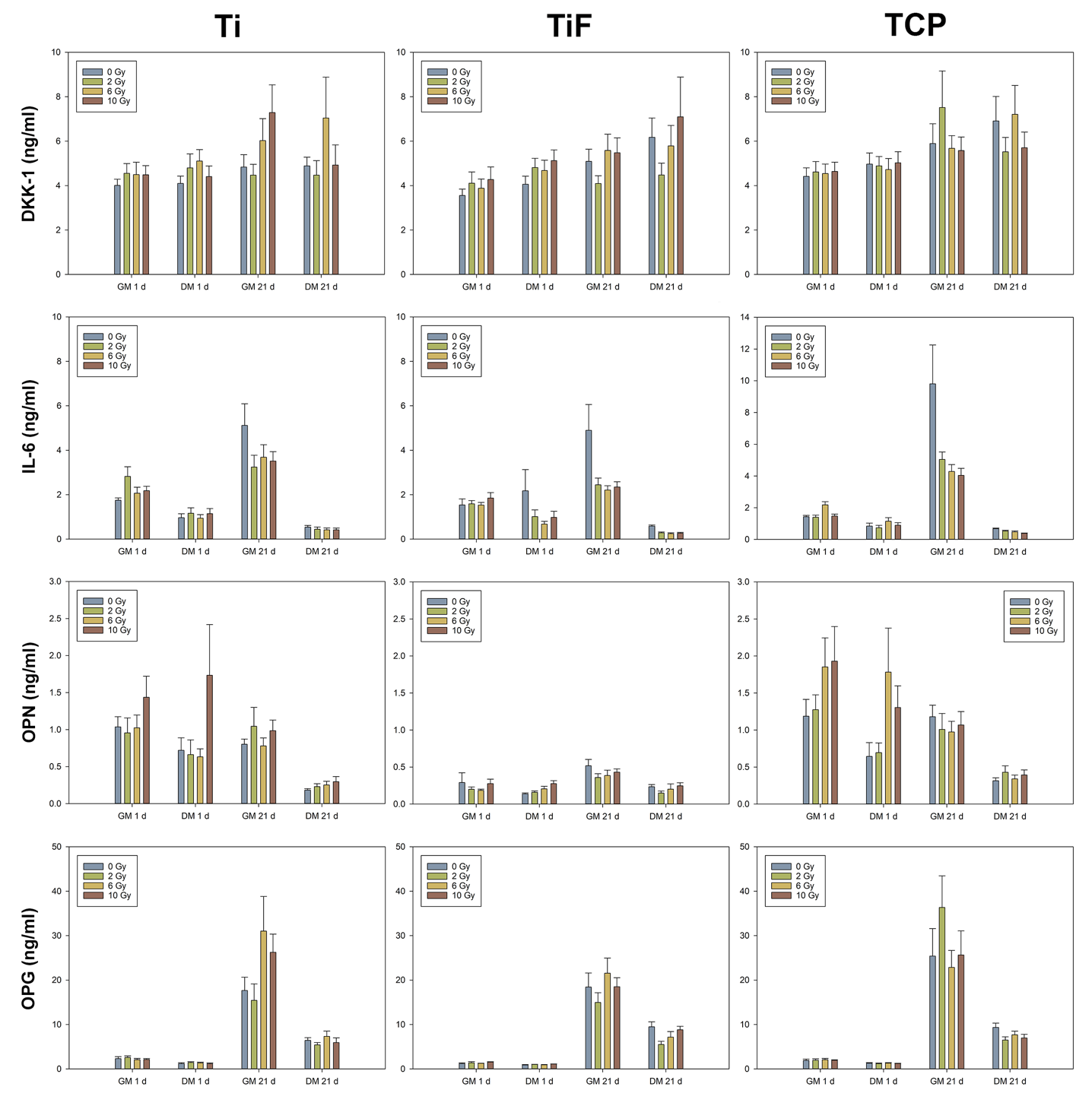


Figure S2. Concentrations of osteogenic biomarkers measured in the cell-culture medium at day 1 and 21 post-irradiation.
